# Supplementary material for: The landscape of biomedical research
Source: Patterns (N Y). 2024 Apr 9;5(6):100968. doi: 10.1016/j.patter.2024.100968 (PMC11240179; doi:10.1016/j.patter.2024.100968)
Supplement: Document S1. Figures S1–S13 and Tables S1–S5 [file mmc1.pdf]

Patterns, Volume *5*

Supplemental information

The landscape of biomedical research

Rita González-Márquez, Luca Schmidt, Benjamin M. Schmidt, Philipp Berens, and Dmitry Kobak

A Appendix

A.1 Supplementary Tables

Table S1: Percentage of abstracts mentioning various machine learning methods (as in Figure 4a) in each region of the embedding with high fraction of abstracts mentioning 'machine learning' (Figure 4b). Percentages above 4% in bold. Rows ordered by the number of papers in the region. Columns ordered by the average percentage across regions. Abbreviations as in Figure 4.

#	Region	NN	Clustering	DL	SVM	CNN	PCA	RF	LR	DR	FA
1	EEG signals	**6.1**	1.7	1.9	3.3	2.1	1.1	0.8	0.8	0.4	0.2
2	Sequencing	1.5	**5.8**	1.0	0.9	0.7	0.4	0.6	0.3	0.3	0.1
3	Image segmentation	**9.5**	2.4	**7.3**	2.5	**7.6**	0.9	1.0	0.5	0.2	0.1
4	ML algorithms	**14.7**	**5.7**	**4.3**	2.9	**5.1**	1.1	0.6	0.5	0.9	0.2
5	Mass spectometry	1.8	1.2	0.2	1.5	0.2	**4.9**	0.4	1.1	0.2	0.5
6	Healthcare data	1.7	0.8	1.6	0.6	0.6	0.0	0.2	0.0	0.0	0.0
7	Cancer biomarkers	0.5	**4.9**	0.2	1.0	0.1	1.1	0.8	0.3	0.1	0.1
8	Protein structure	**5.5**	3.5	1.9	**4.6**	1.0	0.7	1.7	1.4	0.3	0.1
9	Computational chemistry	1.9	0.4	0.3	0.1	0.2	0.1	0.1	0.2	0.1	0.0
10	Tumor imaging	3.3	1.2	2.8	3.6	2.0	0.7	2.6	1.3	0.3	0.1
11	Microbiome	0.1	2.9	0.0	0.2	0.0	1.2	1.5	0.9	0.0	0.1
12	Covid-19 tweets	1.5	2.1	1.9	0.7	0.4	0.2	0.8	0.6	0.0	0.0

Table S2: kNN accuracy of different BERT-based models. This comparison used a subset of the data (training set size: 990,000 labeled papers; test set size: 10,000 labeled papers). For comparison, the kNN accuracy values for the TF-IDF and SVD ($d = 300$) representations measured on the same subset were 61.0% and 54.8% respectively.

	Average	`[CLS]`	`[SEP]`
BERT	57.1%	50.4%	53.4%
SciBERT	62.1%	57.0%	60.9%
BioBERT	64.0%	62.7%	65.0%
PubMedBERT	64.4%	60.4%	**67.7%**
SBERT	64.5%	60.7%	62.2%
SPECTER	64.6%	63.9%	64.7%
SciNCL	65.9%	64.6%	64.6%
SimCSE	57.0%	53.2%	52.1%

Table S3: kNN accuracy of t-SNE representations of different BERT-based models. The same experimental setup as in Table S2. For comparison, the accuracy of t-SNE of the TF-IDF representation (after SVD) was 49.9%.

	Average	`[CLS]`	`[SEP]`
BERT	46.0%	36.3%	40.6%
SciBERT	52.3%	43.4%	48.8%
BioBERT	54.7%	51.1%	56.5%
PubMedBERT	53.2%	45.5%	**60.8%**
SBERT	60.2%	56.3%	56.7%
SPECTER	58.4%	59.2%	59.3%
SciNCL	60.7%	59.1%	59.4%
SimCSE	46.9%	42.4%	40.3%

Table S4: kNN accuracy of label prediction using different transformations of the PubMedBERT representation and two different metrics for finding nearest neighbors. This experiment used test set size 500, smaller than in Table 1.

	Euclidean	Cosine
**Raw**	**67.8%**	**67.8%**
Centered	67.8%	67.4%
Whitened	64.2%	65.4%

Table S5: Runtimes for different analyses.

**Step**	**Time**
Parsing XML	10 h
PubMedBERT representation	74 h
TF-IDF representation	1 h
Truncated SVD of TF-IDF	4 h
t-SNE affinities for PubMedBERT	101 min
t-SNE affinities for TF-IDF	78 min
t-SNE optimization, 750 iter.	126 min
t-SNE optimization, 2250 iter.	390 min
kNNs for 1k papers, BERT	32 min
kNNs for 1k papers, BERT labeled	7 min
kNNs for 1k papers, TF-IDF	150 min
kNNs for 1k papers, TF-IDF labeled	12 min
kNNs for 1k papers, t-SNE	7 min
kNNs for 1k papers, t-SNE labeled	2 min
Table 1	~35 h
Table 2	~91 h
Figure 3c	~20 h
Table S2 (BERT computations)	~30 h
Table S3	~7 h
Table S4	~20 min
All GAMs	~30 min
Gender prediction	6 min

A.2 Supplementary Figures

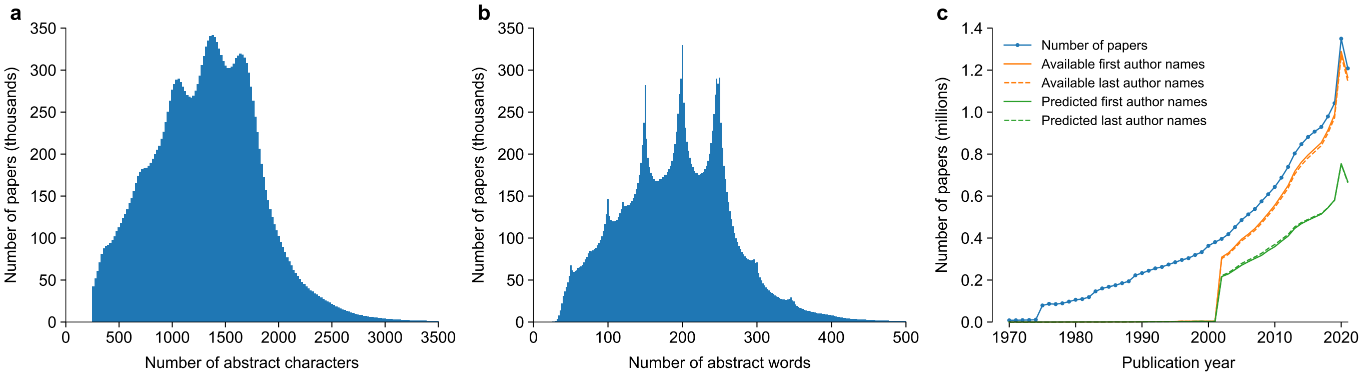

Figure S1: Summary of the PubMed dataset. (a) Distribution of the abstract length in characters. For the distribution of the abstract length over the embedding, see Figure S11. Papers with abstracts shorter than 250 characters were filtered out (see Methods). **(b)** Distribution of the abstract length in words. The smooth peaks visible in panel (a) likely originate from the sharp peaks visible in panel (b), as the journals often specify the maximal allowed abstract length in words (e.g. 150, 200, or 250 words). **(c)** The total number of papers per year, the number of available first/last authors' first names per year, and the number of inferred first/last author genders per year. The amount of available first names increased dramatically after 2003, when PubMed began incorporating more detailed author information into their database (97.4% of available first names are post-2003).

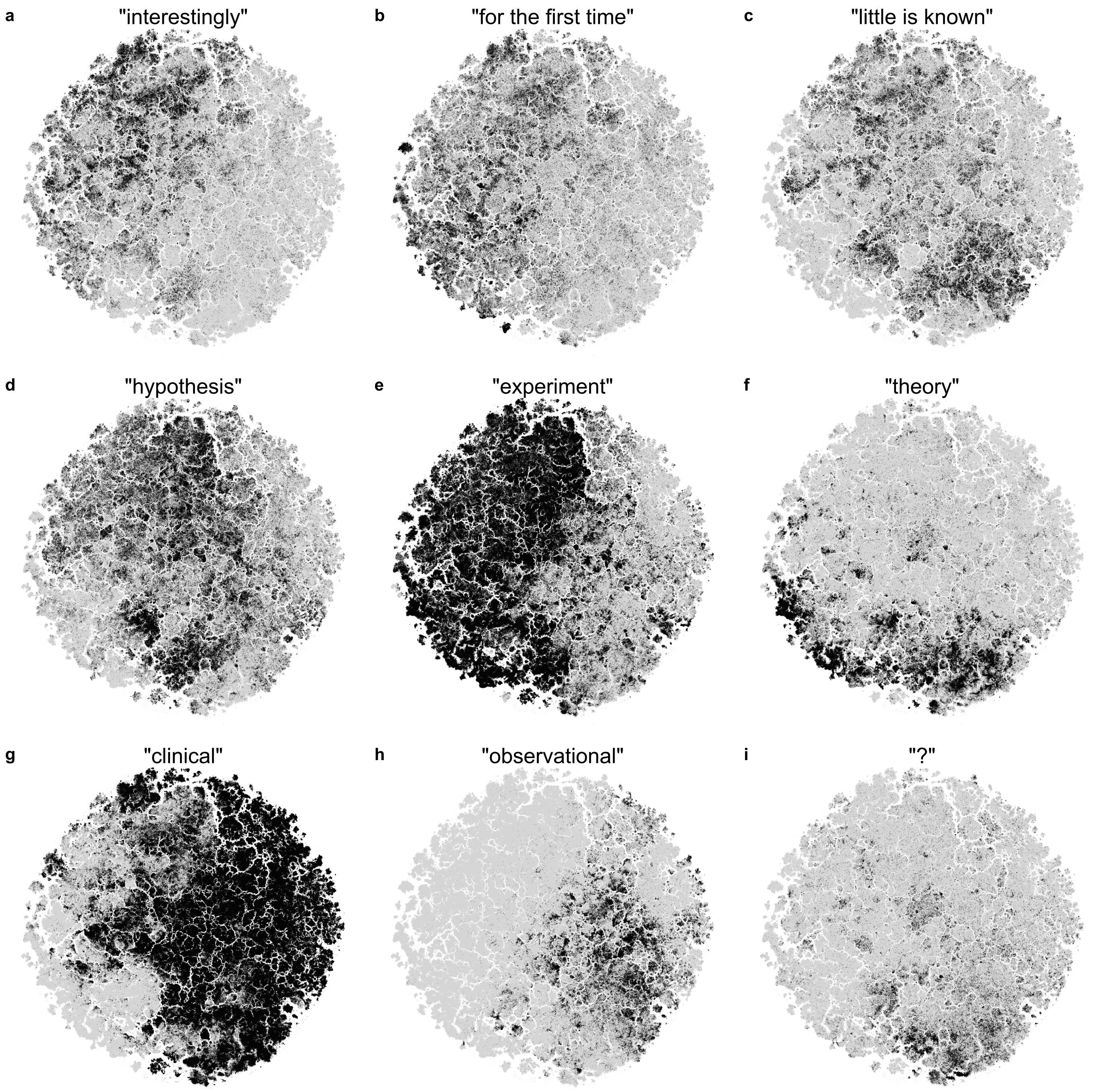

Figure S2: Distribution of some terms and phrases across the biomedical literature. All panels show the embedding based on the PubMedBERT representation, highlighting papers containing particular terms in their abstracts. **(a)** 'interestingly', **(b)** 'for the first time'. Two black islands stand out in the periphery of the embedding: the one in the bottom contains articles reporting new species ('species nova') and the one on the left contains articles reporting novel chemical compounds isolated from living organisms. **(c)** 'little is known', **(d)** 'hypothesis', **(e)** 'experiment', **(f)** 'theory', **(g)** 'clinical', **(h)** 'observational', **(i)** '?' (question mark).

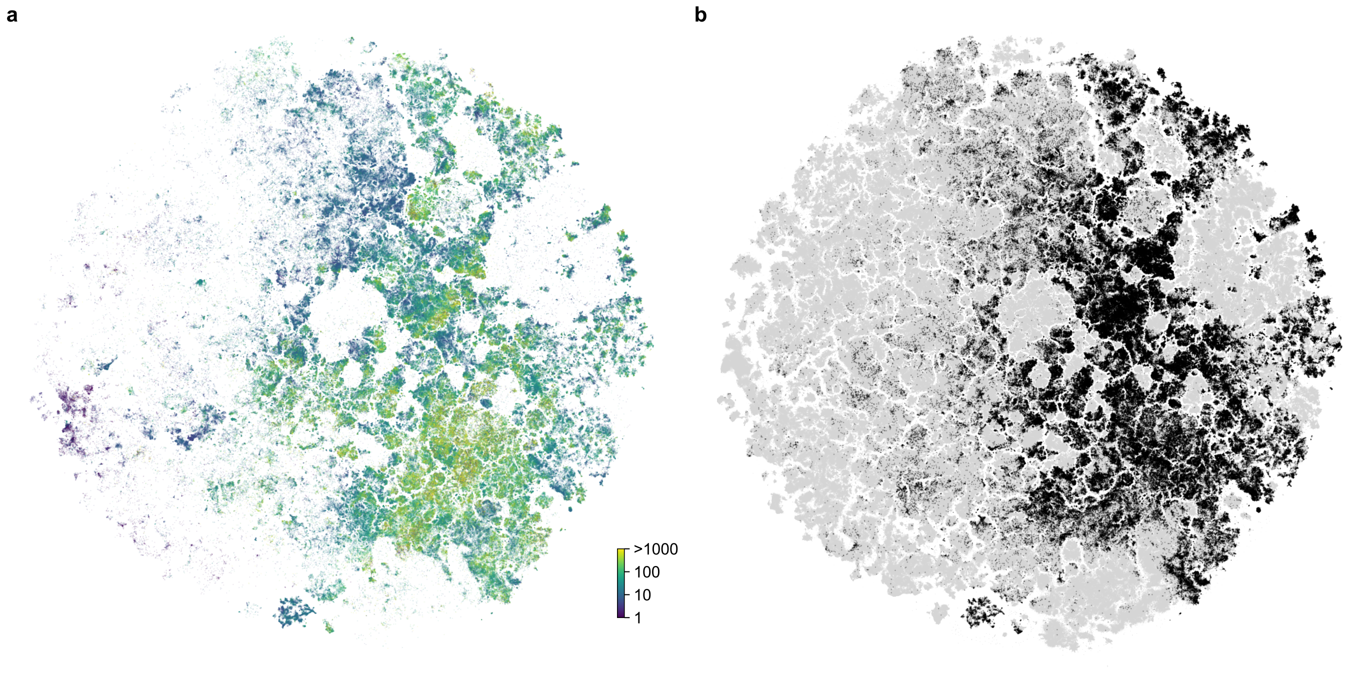

Figure S3: Distribution of reported sample sizes and p-values across the biomedical landscape. (a) Embedding colored by the sample size reported in the abstract. We used the regular expression `n\s?=\s?(\d+)` to extract the reported sample sizes. If an abstract contained several reported sample sizes, we took the first one. Color scale on the log scale, dark: $n = 1$; light: $n \geq 1000$. Papers that did not contain this regular expression in their abstract are not displayed. **(b)** Papers reporting p-values in their abstracts (containing `'p='` or `'p<'` strings, with or without space after 'p') are shown in black.

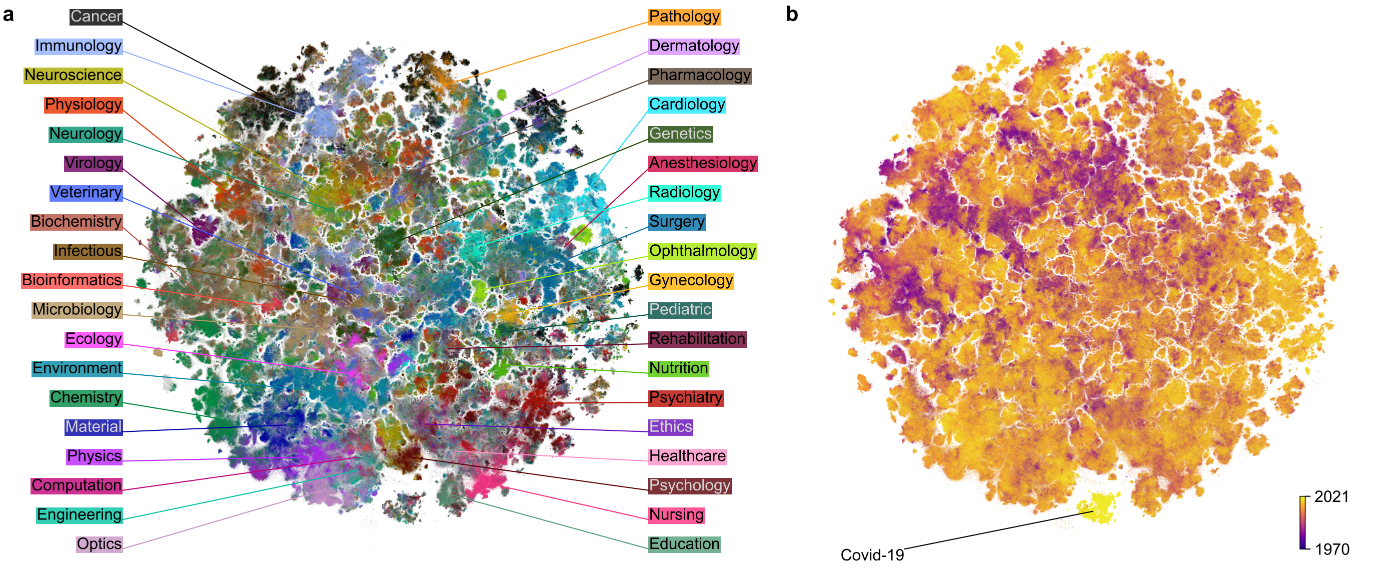

Figure S4: 2D embedding based on the TF-IDF representation of the PubMed dataset. (a) Colored using labels based on journal titles. Unlabeled papers are shown in gray and are displayed in the background. The TF-IDF-based embedding was flipped to orient it similarly to the BERT-based embedding (Figure 1). **(b)** Colored by publication year (dark: 1970 and earlier; light: 2021).

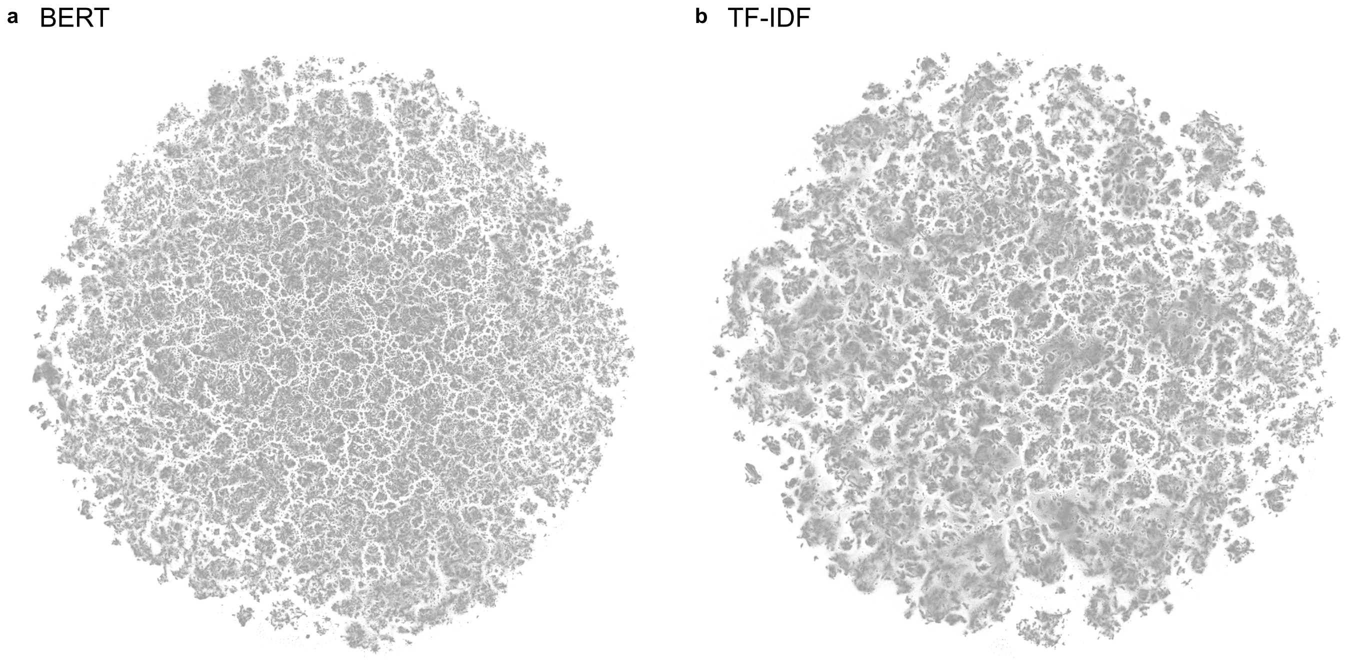

Figure S5: Fine cluster structure in the PubMed embeddings. All points shown in gray to emphasize the cluster structure. **(a)** The embedding based on the PubMedBERT representation. **(b)** The embedding based on the TF-IDF representation.

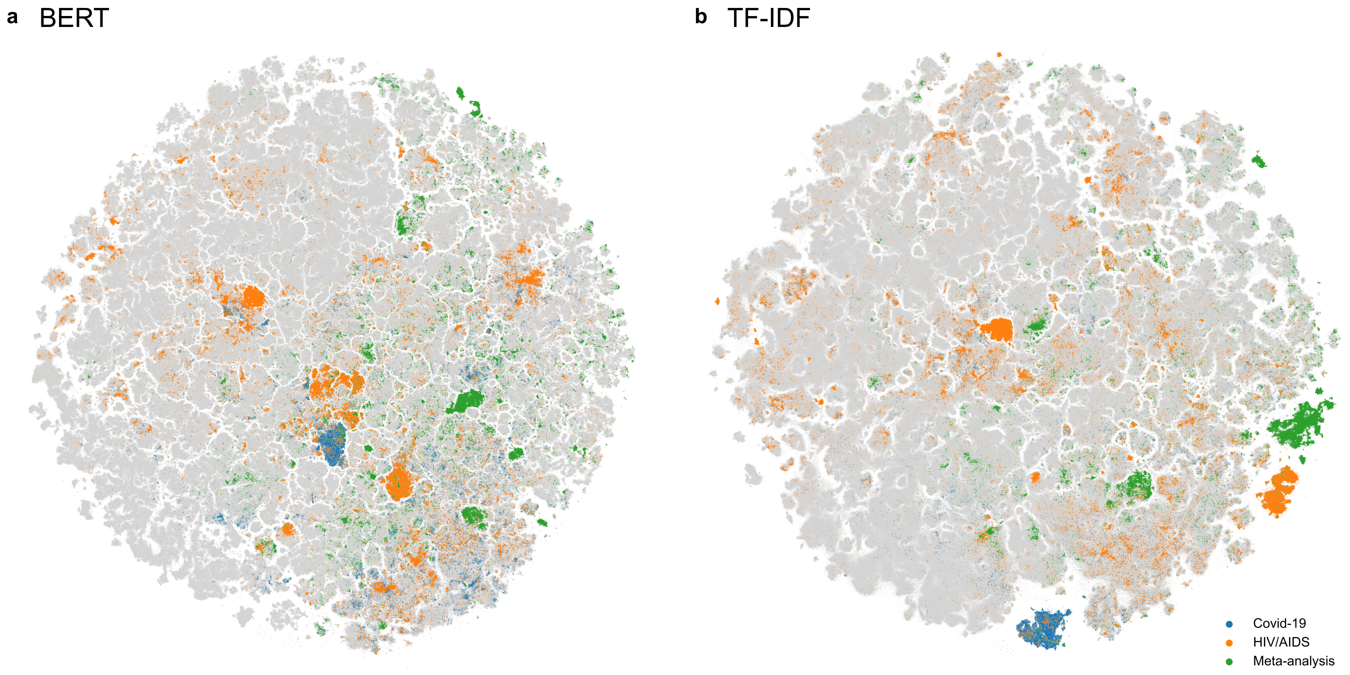

Figure S6: Isolated subcorpora in the PubMed embeddings. Three sets of papers analyzed in Table 2 (Covid-19, HIV/AIDS, meta-analysis) highlighted in both embeddings. **(a)** PubMedBERT-based embedding. **(b)** TF-IDF-based embedding. In the TF-IDF-based embedding, the Covid cluster appeared more separated from the rest of the embedding, and included a larger fraction of Covid papers (86.7%), compared to the BERT-based embedding. Similarly, meta-analysis papers and HIV papers were grouped together and isolated stronger than in the BERT-based embedding. This suggests that TF-IDF representation is more sensitive to the presence of specific keywords than the BERT representation, which is more faithful to semantic similarity between fields (e.g. between Covid papers and the literature on other respiratory diseases).

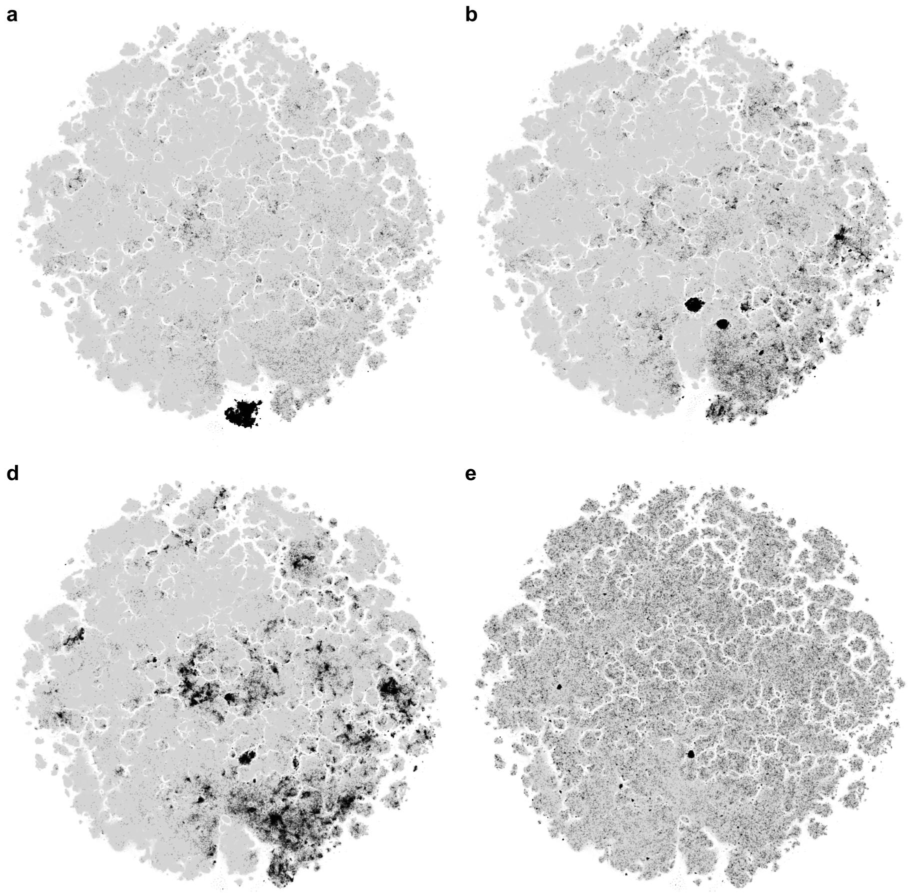

Figure S7: Covid-19 island ablation experiment. The TF-IDF data were altered in several ways and the different versions were used to obtain t-SNE embeddings (following SVD and using the same pipeline as described in the Methods). Papers highlighted in black are Covid papers, unless otherwise stated; non-Covid papers are shown in grey in the background. **(a) Unaltered TF-IDF.** The same embedding as in Figure S4. **(b) TF-IDF without the 'covid' feature.** We eliminated the feature corresponding to the word 'covid' from the TF-IDF data. The Covid island disappeared but the Covid papers were still grouped together in two clear clusters. **(c) TF-IDF without Covid-related features.** We eliminated several Covid-related features ('covid', '19', 'coronavirus', 'sars', 'cov', and '2019') from the TF-IDF data. Here the Covid papers were spread out more. **(d–e) TF-IDF with Covid-related features shuffled.** We shuffled the rows of the TF-IDF submatrix with six columns corresponding to the Covid-related features listed above. The other columns were kept intact. This procedure assigns Covid-related features to random papers in the dataset. In the resulting embedding, the Covid papers were spread out similarly to panel (c). In panel (e) we show the same embedding but highlight the random papers that got assigned the Covid-related features after shuffling. These papers are spread out much more uniformly compared to the Covid papers in panel (d), meaning that the actual Covid papers had much more in common (in the TF-IDF space) than only the Covid keywords.

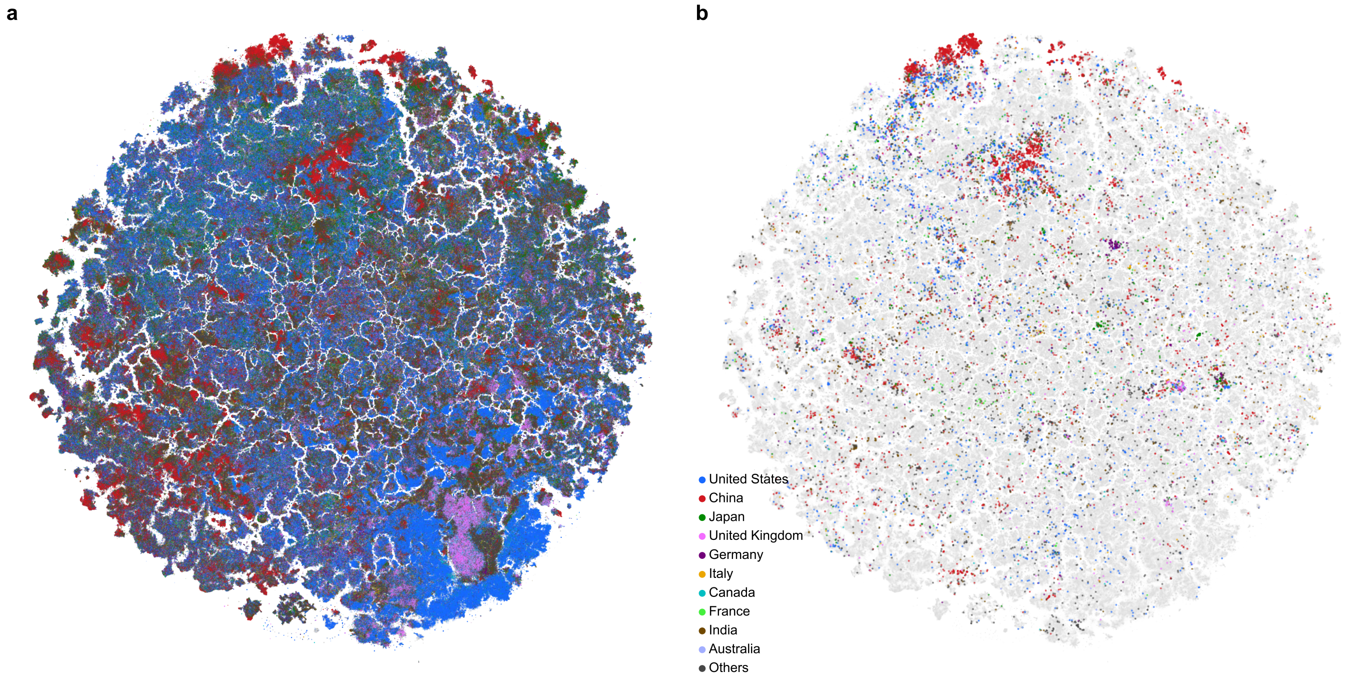

Figure S8: Distribution of affiliation countries across the biomedical landscape. (a) Embedding colored by affiliation country of the first author. Top 10 countries by the total number of papers are shown in colors, the rest are shown in dark gray. Papers without country information are shown in light gray and displayed in the background. **(b)** Embedding showing retracted papers (as in Figure 6), colored by their affiliation countries. Several clusters of retracted papers stem mostly from a single country. In some cases, these clusters correspond to a single author involved in a large-scale scientific misconduct (e.g. German cluster: Joachim Boldt; Japanese cluster: Yoshihiro Sato; US cluster: Scott Reuben). In other cases, clusters contain a larger number of papers from many different authors but one single country, which could be an indicator of paper mill activity. Visible separation between the UK and the US papers in social disciplines (lower-right corner) and in some medical islands may be due to differences between the British and the American spelling.

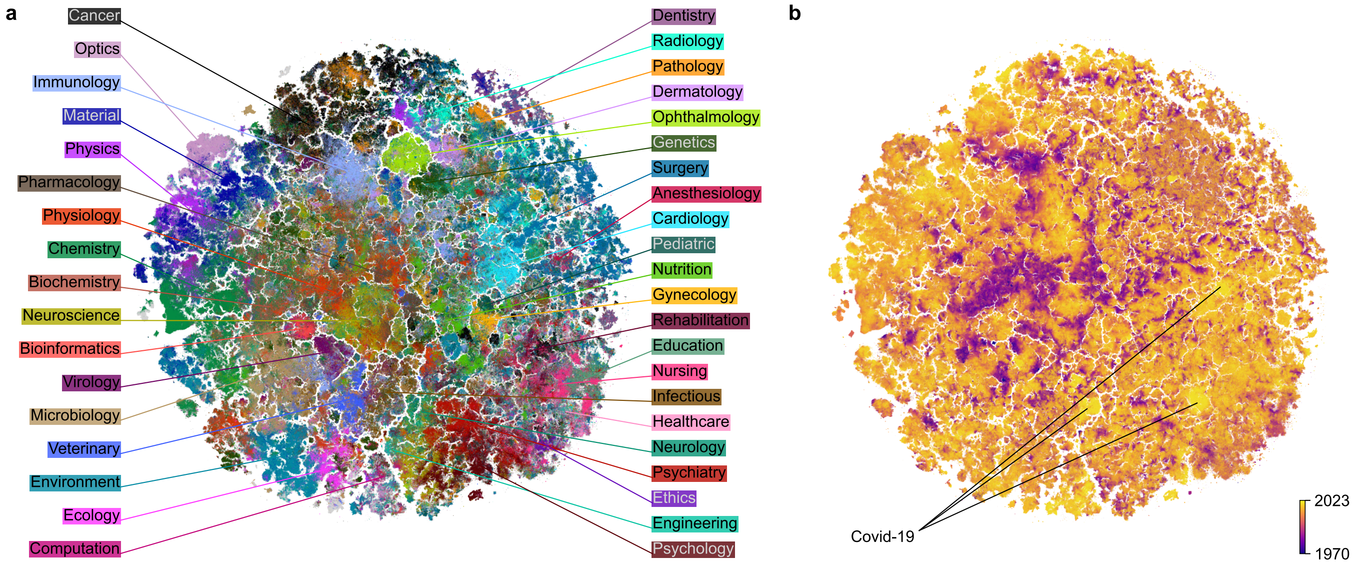

Figure S9: 2D embedding of the updated PubMed dataset. Here we used annual PubMed snapshot that included 2022 and 2023 papers, which were not part of the initial dataset shown in Figure 1. Sample size 23.4 M. **(a)** Colored using labels based on journal titles. Unlabeled papers are shown in gray and are displayed in the background. 'Dentistry' was added as a new label, not present in Figure 1. **(b)** Colored by publication year (dark: 1970 and earlier; light: 2023). The Covid-19 island present in Figure 1 got now divided into three main clusters: the leftmost island contained articles on epidemiology and vaccinology, the lower right island focused on the societal aspects of the pandemic, and the upper right island focused on clinical and medical aspects of Covid-19.

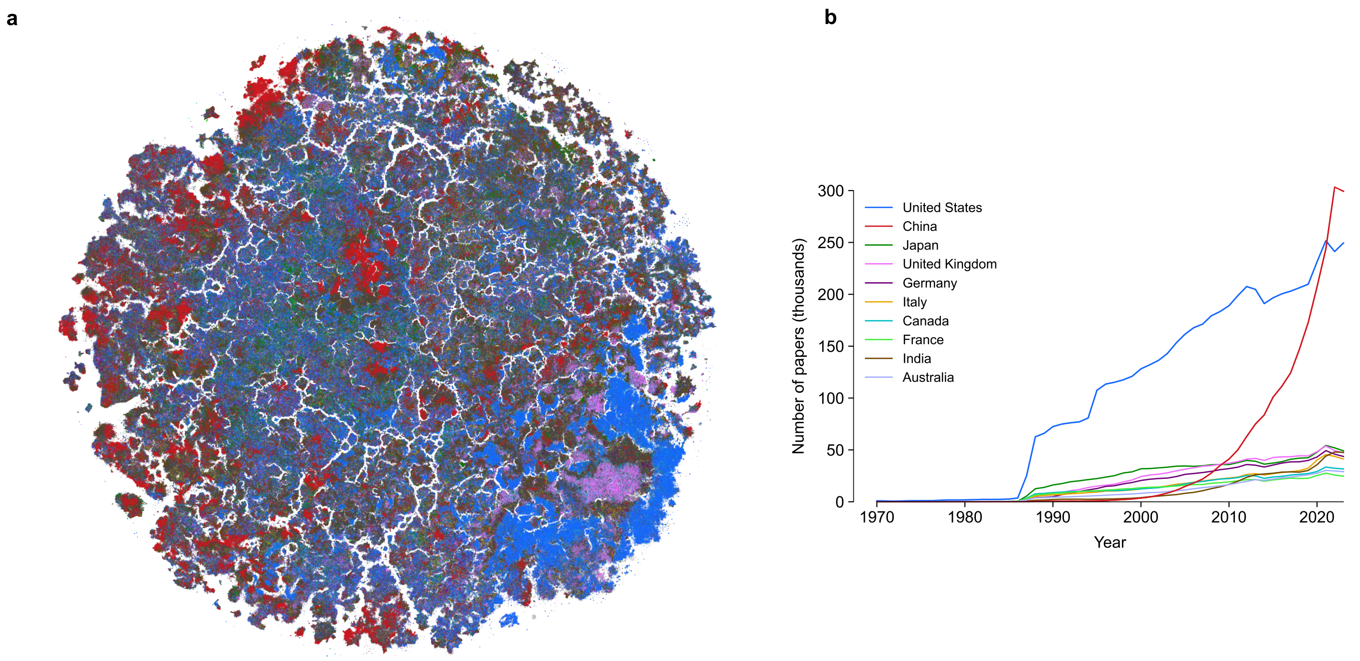

Figure S10: Affiliation countries in the updated PubMed dataset. (a) Updated embedding (Figure S9) colored by the affiliation country of the first author. Top 10 countries by the total number of papers are shown in colors, the rest are shown in dark gray. Papers without country information are shown in light gray and displayed in the background. **(b)** The number of papers from each of the top 10 countries over the years. The number of publications from Chinese institutions has increased exponentially over the last years (Else and Van Noorden, 2021), growing from 3.6 K (1.0% of all publications) in 2000 to 303 K (22.2%) in 2022 and surpassing the US in 2021. Note that the 2023 data in this PubMed snapshot were still incomplete.

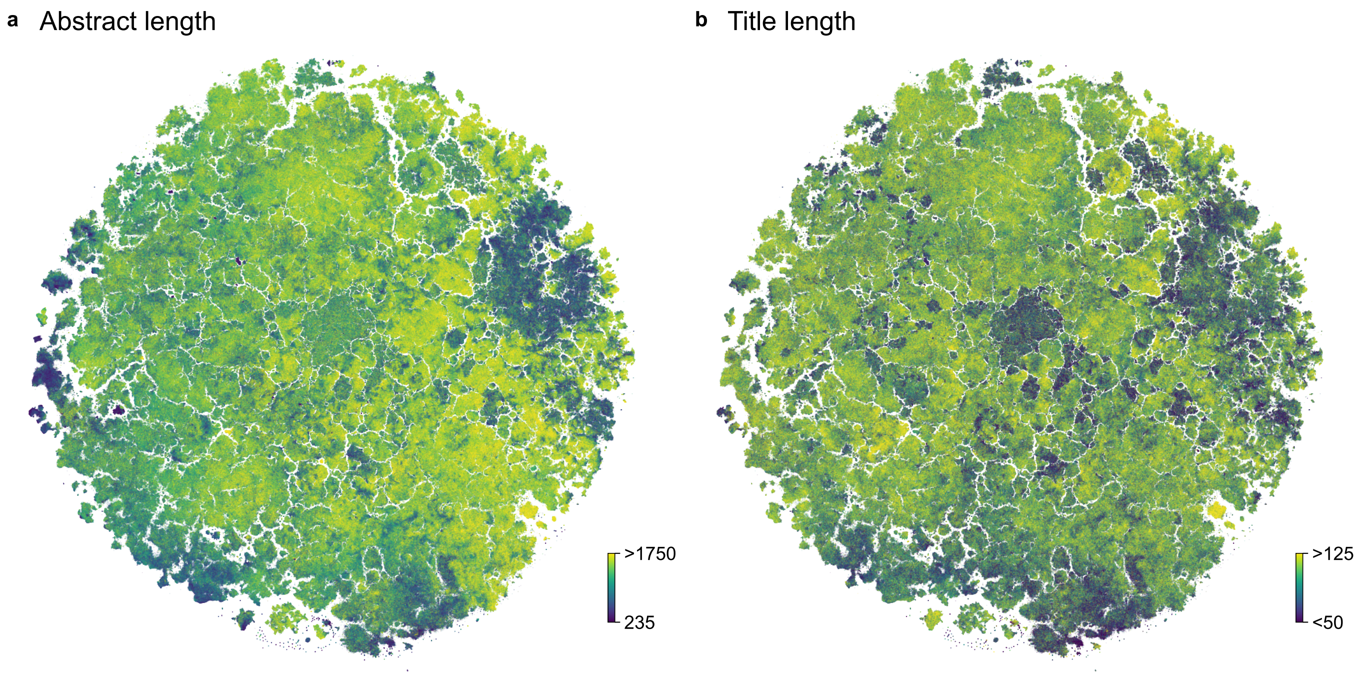

Figure S11: Distribution of abstract and title lengths across the biomedical literature. Both panels show the embedding based on the PubMedBERT representation of the PubMed dataset. **(a)** Colored by the length of the abstract (dark: 235 characters; light: 1750 characters or more). **(b)** Colored by the length of the title (dark: 50 characters or less; light: 125 characters or more).

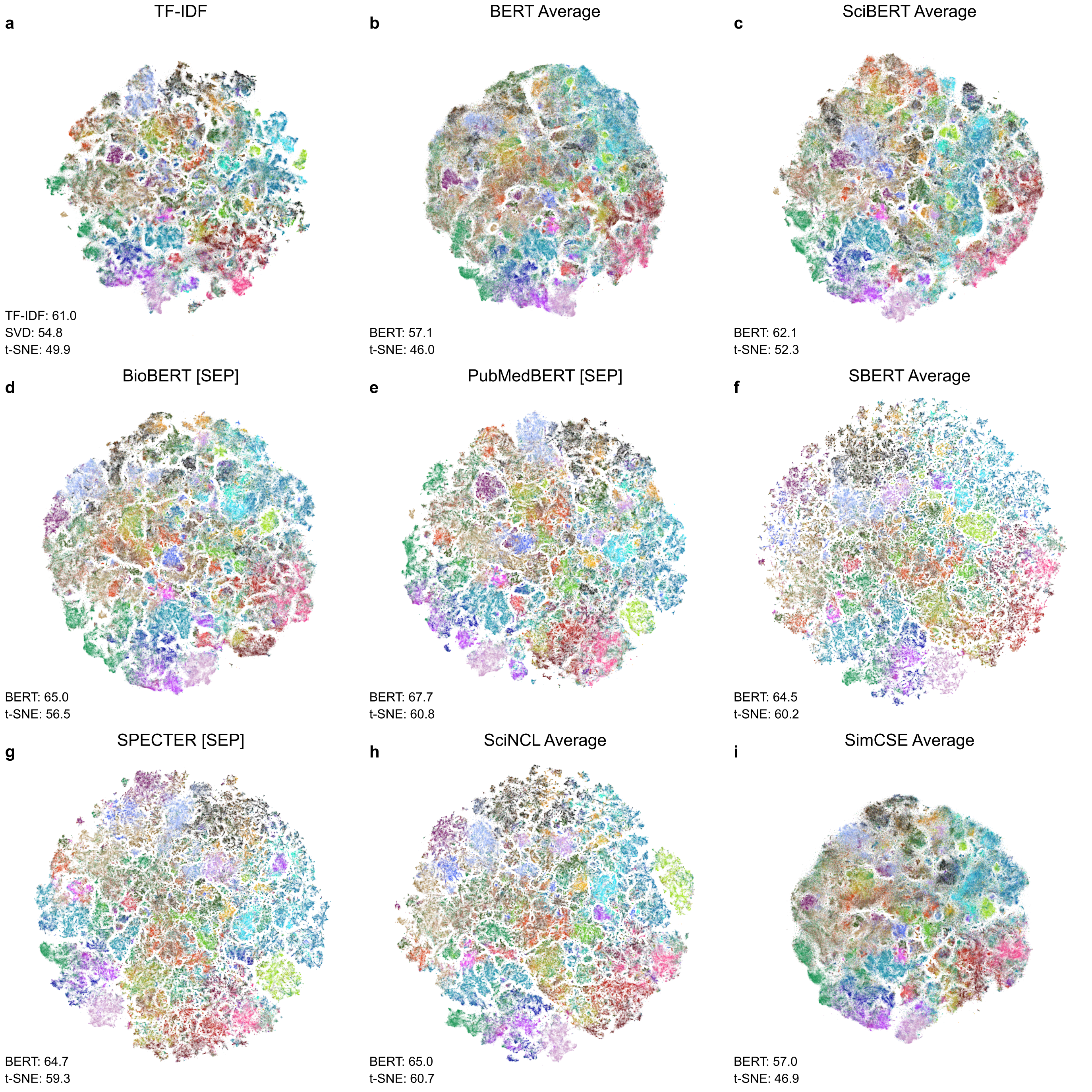

Figure S12: t-SNE embeddings of a subset of the PubMed dataset based on different representations. Subset size: 1,000,000 labeled papers. For each BERT-based model, we chose the two-dimensional embedding based on the representation (average, `[CLS]`, or `[SEP]` token) with the highest kNN accuracy, see Table S3. The kNN accuracies for the high-dimensional and two-dimensional representations are shown in the corner of each panel. The embeddings were flipped to orient them similarly to the embedding of the full dataset (Figure 1). **(a)** TF-IDF (using SVD), **(b)** BERT, **(c)** SciBERT, **(d)** BioBERT, **(e)** PubMedBERT, **(f)** SBERT, **(g)** SPECTER, **(h)** SciNCL, **(i)** SimCSE.

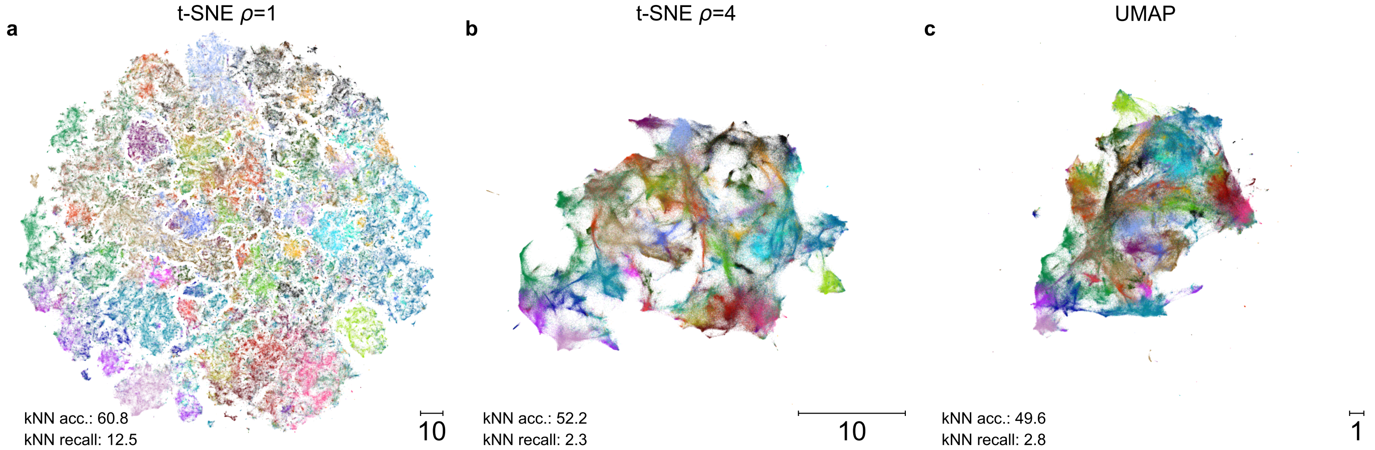

Figure S13: Embeddings of a subset of the PubMed dataset using different neighbor embedding methods. Subset size: 1,000,000 labeled papers. The embeddings were flipped to orient them similarly to the embedding of the full dataset (Figure 1). **(a)** t-SNE without exaggeration ($\rho = 1$). **(b)** t-SNE with exaggeration $\rho = 4$. **(c)** UMAP.
